# Supplementary material for: Cribriform Plate Microenvironment Assembles a Suppressive Myeloid Network during EAE-induced Neuroinflammation
Source: bioRxiv. 2026 Jan 8:2026.01.07.698165. Preprint. [Version 1] doi: 10.64898/2026.01.07.698165 (PMC12803268; doi:10.64898/2026.01.07.698165)
Supplement: Supplement 1 [file NIHPP2026.01.07.698165v1-supplement-1.pdf]

bioRxiv preprint doi: https://doi.org/10.64898/2026.01.07.698165; this version posted January 8, 2026. The copyright holder for this preprint (which was not certified by peer review) is the author/funder, who has granted bioRxiv a license to display the preprint in perpetuity. It is made available under aCC-BY 4.0 International license.

## Supplemental Figure 1 - Strategy to isolate CD11b+CD11c+PDPN+CD11b+ “No contact” and “Post-Contact” populations

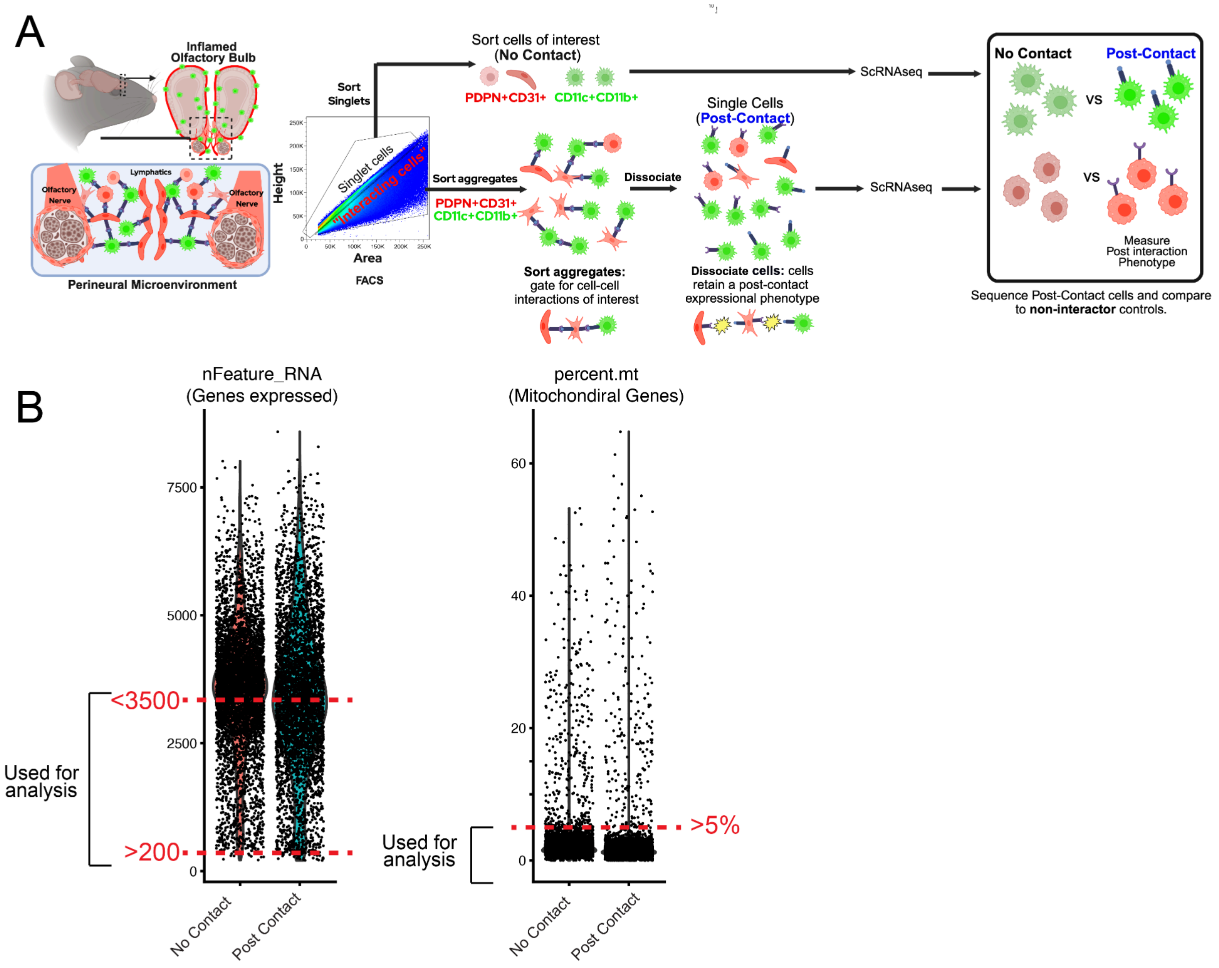

bioRxiv preprint doi: https://doi.org/10.64898/2026.01.07.698165; this version posted January 8, 2026. The copyright holder for this preprint (which was not certified by peer review) is the author/funder, who has granted bioRxiv a license to display the preprint in perpetuity. It is made available under aCC-BY 4.0 International license.

**Supplemental Figure 1 - Strategy to isolate CD11b+CD11c+PDPN+CD11b+ "No contact" and "Post-Contact" populations**

**(A):** Cartoon scheme outlining "PostContact-seq" sorting procedure of cribriform plate + Olfactory bulb tissue preparations from EAE 3.0. Sorted singlets from $CD11c^{+}CD11b^{+}$ (Myeloid cells) and $CD31^{+}PDPN^{+}$ (Meningeal-Lymphatic niche) gates were collected into a no-contact tube. Simultaneously, quadruple positive $CD11c^{+}CD11b^{+}CD31^{+}PDPN^{+}$ (Myeloid cell+Meningeal Lymphatic Niche) aggregating cells were sorted into the "interactor" tube. Prior to scRNAseq, both tubes underwent short liberase treatment to dissociate interacting cells and generate single cell suspensions of post-contact, and sequenced.

**(B):** Cutoffs for filtered cells for differential analysis using the following criteria: Cells have > 200 genes expressed, Cells have < 3,500 genes expressed, Cells have < 5% mitochondrial genes expressed. Strategy limits doublet aggregates at time of sequencing (High nFeatureRNA) and dead cells (High Percentage Mt).

bioRxiv preprint doi: https://doi.org/10.64898/2026.01.07.698165; this version posted January 8, 2026. The copyright holder for this preprint (which was not certified by peer review) is the author/funder, who has granted bioRxiv a license to display the preprint in perpetuity. It is made available under aCC-BY 4.0 International license.

**Supplemental Figure 2. Analysis of PD-1⁺ immune subsets in isolated cribriform plate cell suspensions**

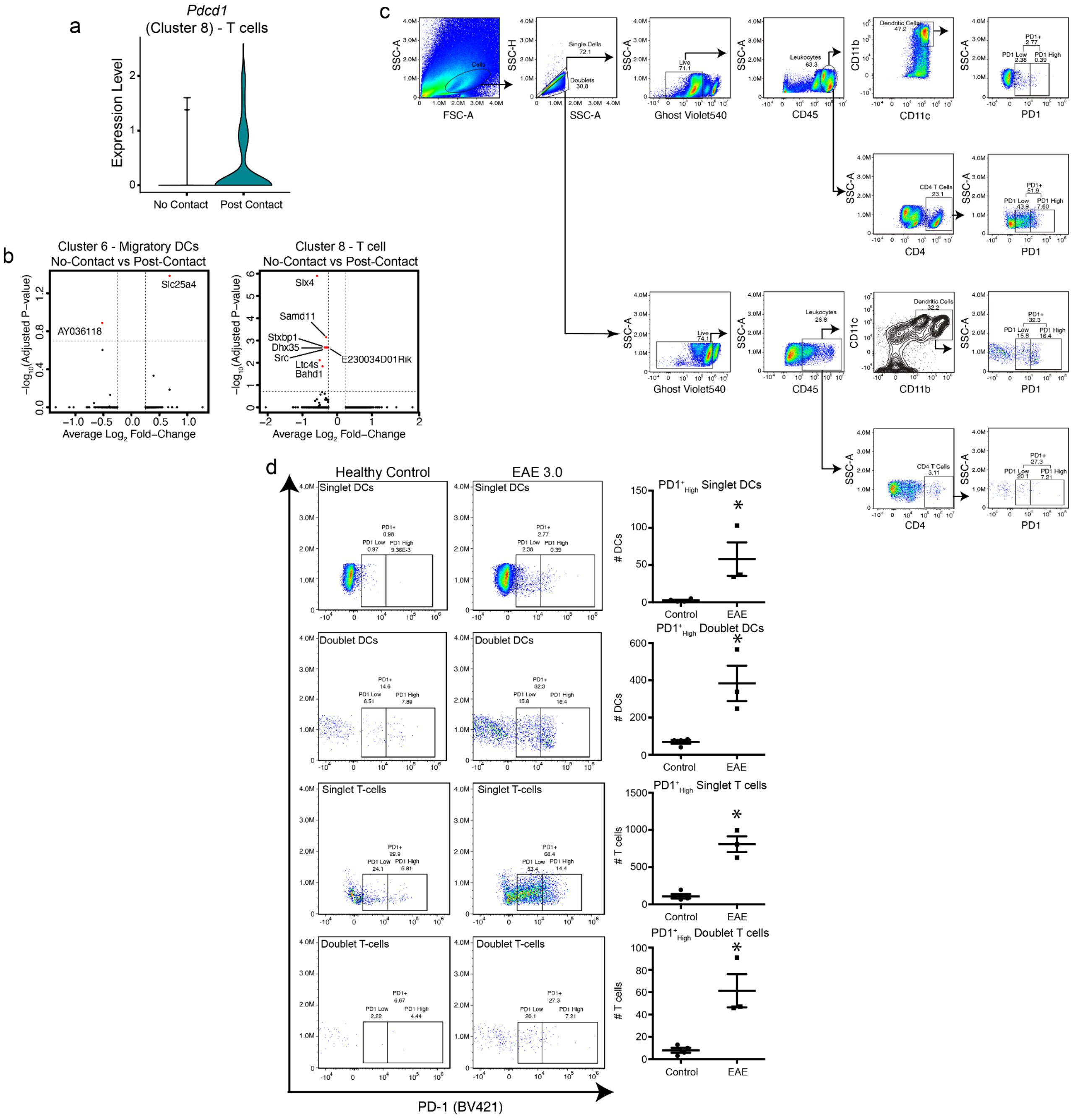

bioRxiv preprint doi: https://doi.org/10.64898/2026.01.07.698165; this version posted January 8, 2026. The copyright holder for this preprint (which was not certified by peer review) is the author/funder, who has granted bioRxiv a license to display the preprint in perpetuity. It is made available under aCC-BY 4.0 International license.

**Supplemental Figure 2. Analysis of PD-1$^{+}$ immune subsets in isolated cribriform plate cell suspensions**

**(A)** Violin plot showing *Pdcd1* (PD-1) expression in T cells (Cluster 8) stratified by inferred cell-cell interactions, with higher expression observed in post contact T cells.

**(B)** Volcano plots of differential gene expression between post-contact and no-contact cells in Cluster 6 migratory dendritic cells (left) and Cluster 8 T cells (right)

**(C)** Flow cytometry gating strategy for identifying singlet and doublet populations of CD45$^{+}$ leukocytes, dendritic cells (CD11c$^{+}$), and CD4$^{+}$ T cells, followed by assessment of PD-1 expression across subsets.

**(D)** Representative flow plots and quantification of PD-1$^{high}$ singlet and doublet dendritic cells and T cells in healthy control and EAE day 3.0 mice. EAE mice show significantly elevated PD-1$^{high}$ populations across all subsets, with the largest increase observed in doublet T cells, suggesting enhanced immune interaction and checkpoint activation during neuroinflammation. Singlet DCs (p=0.0322), Doublet DCs (p=0.0084), Singlet T cells (p=0.0005), Doublet T cells (p=0.0007). Unpaired two-tailed t test. Data are represented as mean ± SEM

bioRxiv preprint doi: https://doi.org/10.64898/2026.01.07.698165; this version posted January 8, 2026. The copyright holder for this preprint (which was not certified by peer review) is the author/funder, who has granted bioRxiv a license to display the preprint in perpetuity. It is made available under aCC-BY 4.0 International license.

**Supplemental Figure 3. Cell cluster characterization of "post contact" $CD11b^{+}CD11c^{+}$ $PDPN^{+}CD31^{+}$ interacting cells**

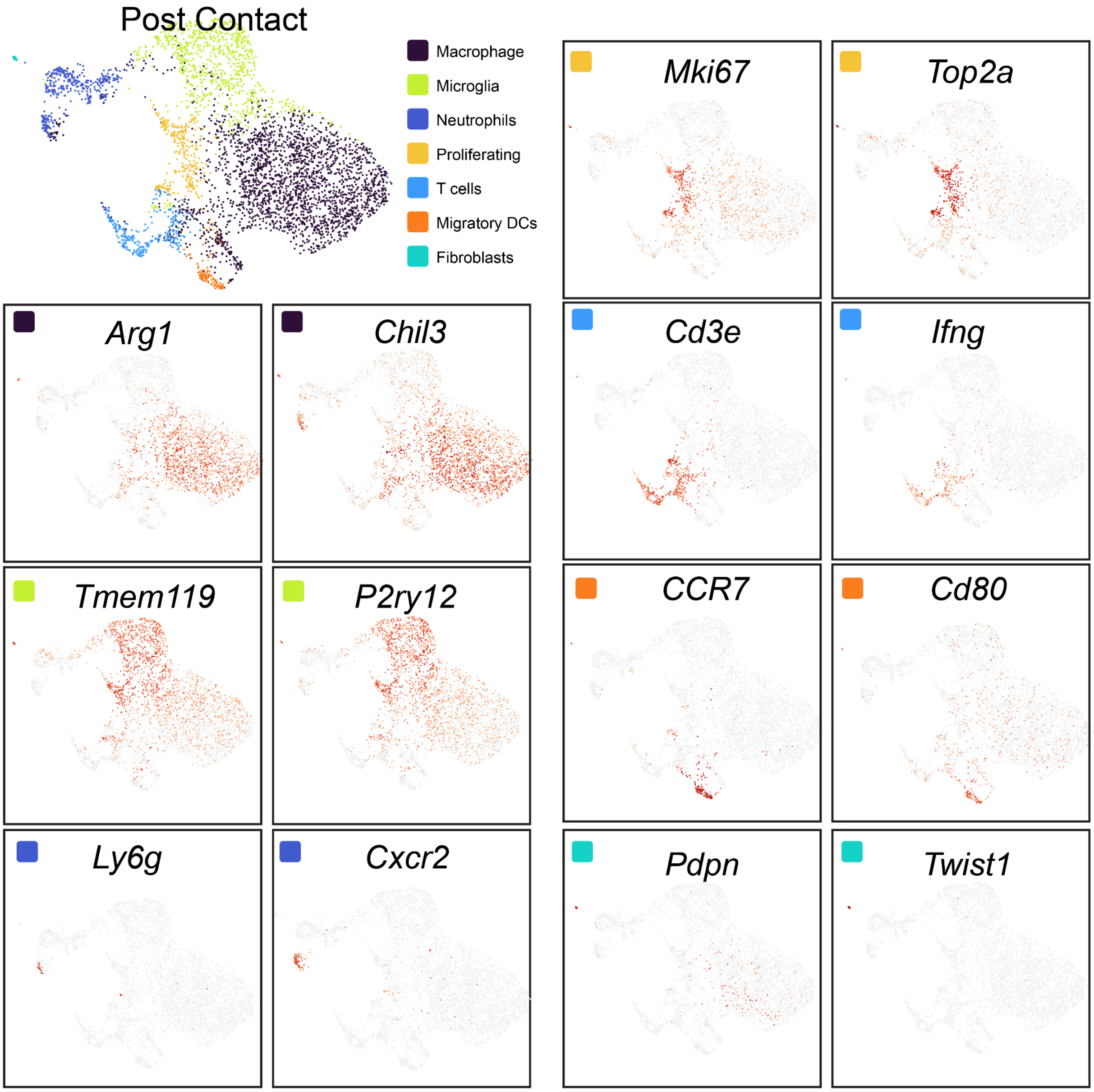

bioRxiv preprint doi: https://doi.org/10.64898/2026.01.07.698165; this version posted January 8, 2026. The copyright holder for this preprint (which was not certified by peer review) is the author/funder, who has granted bioRxiv a license to display the preprint in perpetuity. It is made available under aCC-BY 4.0 International license.

**Supplemental Figure 3. Cell cluster characterization of "post contact" CD11b$^+$CD11c$^+$ PDPN$^+$CD31$^+$ interacting cells**

UMAP projection (top left) shows major post contact cell types isolated, including macrophages (purple), microglia (green), neutrophils (blue), T cells (orange), proliferating cells (light blue), migratory dendritic cells (yellow-orange), and fibroblasts (aqua). Feature plots highlight expression of *Arg1*, *Ly6c2*, *Ccr2*, *Chil3* (CHI3L3), and *Pdpn*, identifying infiltrating monocyte-derived, alternatively activated macrophages, distinct from T cells (*Cd3e*, *Ifng*), DCs (*CCR7*, *Cd80*), microglia (*Tmem119*, *P2ry12*), neutrophils (*Ly6g*, *Cxcr2*), and proliferating cells (*Mki67*, *Top2a*). Gene ontology enrichment analysis (bottom) demonstrates that these post contact macrophages are enriched for pathways in Fcγ receptor and complement receptor signaling, negative regulation of IL-12 production, homotypic cell-cell adhesion, and hemostasis.

bioRxiv preprint doi: https://doi.org/10.64898/2026.01.07.698165; this version posted January 8, 2026. The copyright holder for this preprint (which was not certified by peer review) is the author/funder, who has granted bioRxiv a license to display the preprint in perpetuity. It is made available under aCC-BY 4.0 International license.

**Supplemental Figure 4. Accumulation of $CCR2^+$ immune cells in $PDPN^+$ cribriform regions during EAE**

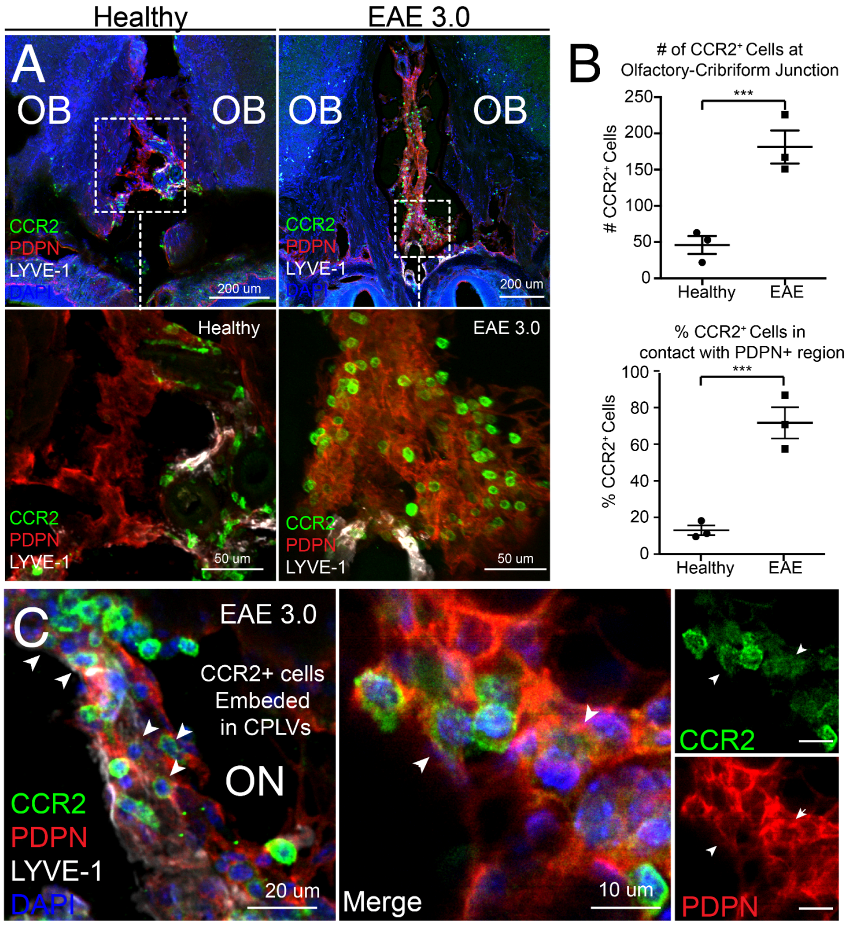

bioRxiv preprint doi: https://doi.org/10.64898/2026.01.07.698165; this version posted January 8, 2026. The copyright holder for this preprint (which was not certified by peer review) is the author/funder, who has granted bioRxiv a license to display the preprint in perpetuity. It is made available under aCC-BY 4.0 International license.

**Supplemental Figure 4. Accumulation of $CCR2^+$ immune cells in $PDPN^+$ cribriform regions during EAE**

**(A)** Immunofluorescence images of healthy and EAE 3.0 olfactory bulbs (OBs), showing increased $CCR2^+$ macrophage accumulation at the olfactory-cribriform junction. Insets show higher magnification of boxed areas with increased $CCR2^+$ cell localization to PDPN-rich regions in EAE.

**(B)** Quantification of $CCR2^+$ cell numbers and their proportion in contact with PDPN-rich zones at the olfactory-cribriform interface. EAE mice show significantly more $CCR2^+$ cells and higher PDPN-association. Unpaired two-tailed t test. Data are represented as mean ± SEM ($p$ = 0.0027).

**(C)** High-magnification image showing $CCR2^+$ cells (green) embedded within cribriform plate lymphatic vessels (CPLVs, marked by LYVE-1, white) and adjacent to $PDPN^+$ region (red) at the olfactory nerve (ON) interface in EAE 3.0 mice. Arrowheads indicate $CCR2^+$ cells associated with CPLVs. Unpaired two-tailed t test. Data are represented as mean ± SEM ($p$ = 0.0064)

bioRxiv preprint doi: https://doi.org/10.64898/2026.01.07.698165; this version posted January 8, 2026. The copyright holder for this preprint (which was not certified by peer review) is the author/funder, who has granted bioRxiv a license to display the preprint in perpetuity. It is made available under aCC-BY 4.0 International license.

**Supplemental Figure 5. Graphical Summary**

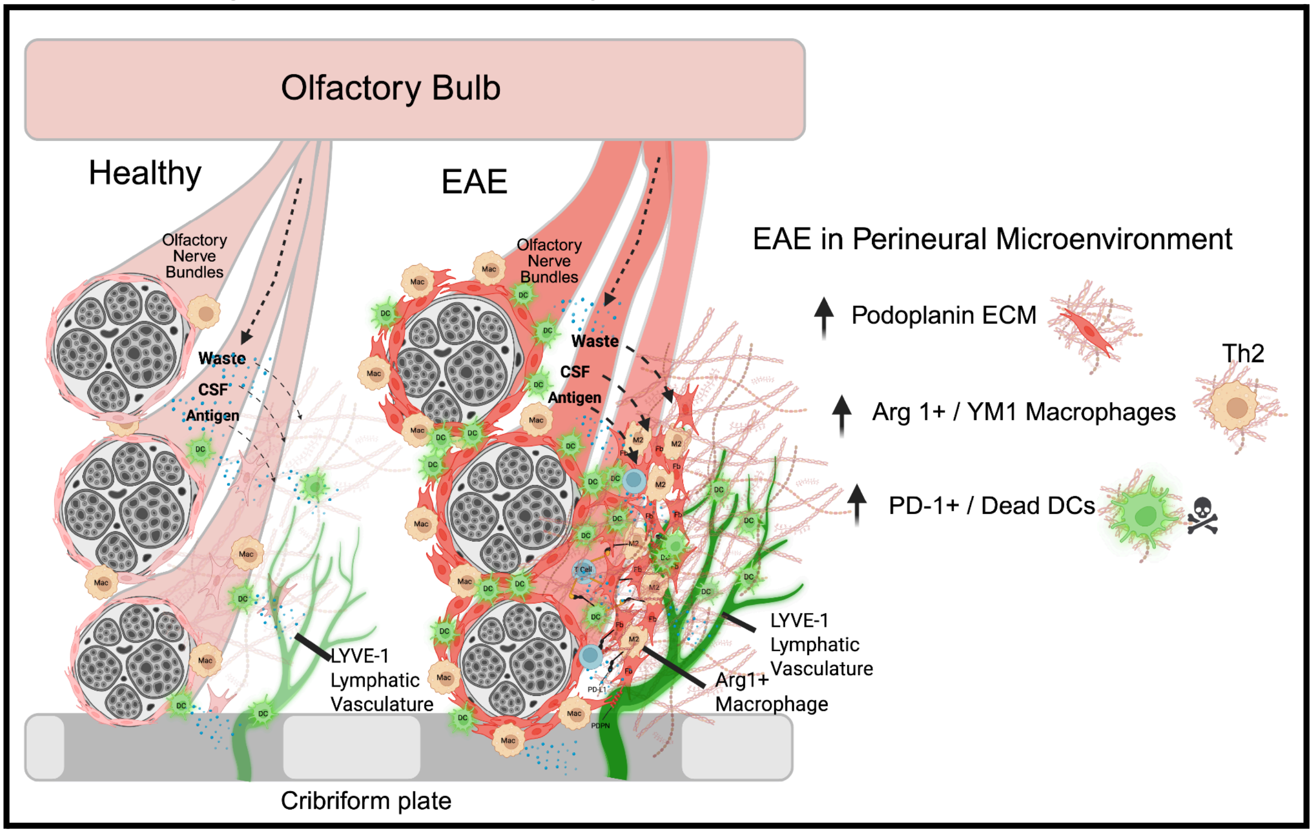


**Supplemental Figure 5. Graphical Summary.** Perineural Microenvironment (PME) has Expanded Immune-Stromal-Lymphatic Niche During EAE. In the healthy state the PME of olfactory nerve bundles (ON) have lower levels of myeloid cells and lymphatics but still have access to draining cerebrospinal fluid (CSF), waste, antigen, and immune cells like DCs. During EAE neuroinflammation the PME becomes remodeled with higher myeloid cell accumulation within PDPN+ regions: fibroblasts, lymphatics, ECM, and macrophages. This creates an assembled immunoregulatory environment around olfactory nerve bundles where tolerogenic myeloid cells engage in cell-cell interactions alongside nerve bundles and cribriform lymphatics.
